# How disease threat affects the purchase intention of ultra-processed foods among high-pressure workers

**DOI:** 10.3389/fnut.2025.1574726

**Published:** 2025-07-23

**Authors:** Linli He, Dajun Yang, Yifan Huang

**Affiliations:** ^1^Department of Pathology, Affiliated Hospital of North Sichuan Medical College, Nanchong, China; ^2^Institute of Basic Medicine, North Sichuan Medical College, Nanchong, China; ^3^The Primary Health Development Research Center of Sichuan Province, North Sichuan Medical College, Nanchong, Sichuan Province, China

**Keywords:** disease threat, high-pressure workers, perception of health, ultra-processed foods, fear, purchase intention

## Abstract

**Introduction:**

The continuous outbreak of global epidemics has highlighted the impact of disease threats on consumer behavior. While prior research has examined the influence of disease threats on consumer decisions, few studies have explored their specific effect on the purchase intention of ultra-processed foods among high-pressure workers. This study examines how disease threats affect the purchase intention of ultra-processed foods among this group.

**Methods:**

We conducted three experiments with 828 participants, employing Process Model 4 and Model 1 to analyze the relationship between disease threats, health perception, fear, and the intention to purchase ultra-processed foods.

**Result:**

Our findings indicate that disease threats significantly reduce the purchase intention of ultra-processed foods among high-pressure workers, with health perception serving as a mediator of this relationship. Additionally, fear interacts with disease threats to further shape purchase behavior.

**Conclusion:**

This research offers novel insights into the consumption patterns of high-pressure workers under disease threats, providing theoretical guidance for public health policies and marketing strategies.

## 1 Introduction

Ultra-processed foods refer to foods that have undergone a series of complex industrial processing methods (1, 2). There is ample evidence that ultra-processed foods are associated with various adverse health outcomes, including obesity, cardiovascular diseases, diabetes, and cancer (3–5). However, in daily life, people have a strong preference for these types of foods. On the one hand, ultra-processed foods are made more delicious through the addition of various ingredients and additives (6, 7). On the other hand, they have a longer shelf life, which reduces economic losses caused by food becoming inedible due to long-term storage (8). However, previous research on ultra-processed foods has mainly focused on health impacts, cognitive functions, food component analysis, food processing technology, and food safety and nutrition (8–10). There has been little attention to the purchase intention of ultra-processed foods among high-pressure workers. High-pressure work environments can lead to psychological health problems such as anxiety and depression (11, 12). In such cases, high-pressure workers may seek quick emotional comfort, and ultra-processed foods may become their choice for relieving stress due to their convenience and taste. However, long-term reliance on these foods may exacerbate health problems, reduce physical immunity, increase the risk of disease, and create a vicious cycle (13, 14). Despite the health risks, the convenience, adaptability to high-pressure work, sensory stimulation, and emotional compensation offered by super-processed foods, along with their bright packaging and low prices, greatly promote their purchase among high-stress workers (15–17). For example, in the study of Lopes Cortes et al. (18), it was found that the willingness to buy ultra-processed food in the high-stress working group was 1.94 times higher than that in the general working group. The global sales data of ultra-processed solid foods and ultra-processed beverages in 2019 show that the per capita consumption of ultra-processed foods in high-income countries is 109.3 kg per year, much higher than that in other countries, reaching 3.4 times and 11.3 times of per capita sales in upper-middle income countries and lower-middle income countries, respectively (19). In recent studies, a large number of researchers have highlighted that disease threats have a significant impact on consumer behavior (20). However, few studies have focused on how disease threats affect the purchase intention of ultra-processed foods among high-pressure workers. The outbreaks of the Marburg virus, monkeypox, and the latest avian influenza virus have posed a significant threat to human life safety worldwide (21, 22). As of November 17, 2024, 20 African countries have reported 12,596 confirmed cases of monkeypox.^1^ The emergence of global epidemics has further intensified the disease threat to high-pressure working groups (23). Previous studies have overlooked the impact of external disease threats on consumer willingness to purchase ultra-processed foods, particularly in high-stress work environments. This neglect is a great pity, as ultra-processed foods have been shown to have significant positive associations with obesity, cardiovascular disease, diabetes, intestinal disease, and psychological disease in multiple studies (24–27). These diseases, as external health threats, may affect consumer perception of ultra-processed foods and purchase decisions (28). Therefore, studying how the disease threat among high-pressure workers affects their purchase intention of ultra-processed foods has great practical significance.

In recent years, disease threats have received widespread attention in the field of marketing. For example, in the study by Yi et al. (29), it was found that the threat of infectious diseases during the epidemic affected consumer sentiment through price factors. In another study, Wu et al. (30) also showed that panic buying occurred after the COVID-19 pandemic, with the primary factor being the threat of a personal health crisis. Through a literature review, we found that previous research has primarily focused on the emergence of new viruses or diseases, consumer behavior, public health, and disease management in patients (29, 31). For instance, the study by Yi et al. (32) showed that the disgust and fear of diseases led consumers to prefer purchasing more familiar products. Although the above studies have focused on the emotions and consumption choices generated by disease threats, they have overlooked the role of disease threats in the consumption of ultra-processed foods. A recent study has highlighted that patients affected by disease threats are more likely to purchase green, organic foods (33). Therefore, we have focused on the impact of disease threats on the purchase intention of ultra-processed foods among high-pressure workers. We will examine the relationship between disease threats and the purchase intention of ultra-processed foods, and investigate the mechanisms that influence this relationship.

To fill the gap in the above research, this study has developed a framework to explore whether and why high disease threat high-pressure workers have a lower purchase intention for ultra-processed foods. This study adopts the Perceived Threat Theory as its guiding framework, positing that in the context of disease threat, the purchase intention of ultra-processed foods among high-pressure workers may be influenced by their perception of health threats (34, 35). Disease threats have been confirmed by numerous studies to influence consumers’ purchasing behavior (36–38). However, previous research has primarily focused on consumer disease threats and consumption behavior, with few studies examining the relationship between disease threats and ultra-processed foods among high-pressure workers. Based on this, we first propose that the disease threat of high-pressure workers has a significant impact on the purchase intention of ultra-processed foods; secondly, we analyze the mediating effect of health perception on the relationship between disease threat and the purchase intention of ultra-processed foods from the perspective of health perception; finally, we explore the moderating effect of fear on the relationship between disease threat and the purchase intention of ultra-processed foods from the perspective of the emotions of high-pressure workers.

This study makes the following contributions to disease threats and consumer behavior. First, this study is one of the early studies to link disease threats, high-pressure worker groups, and ultra-processed foods, making a contribution to the existing literature on disease threats and consumer behavior. Second, this study introduces health perception as a mediator, exploring the mediating effect of health perception on the relationship between the disease threat faced by high-pressure workers and their purchase intention of ultra-processed foods, thereby providing a better understanding of the internal connection between disease threats and consumer behavior. Third, this study examines the moderating effect of fear, analyzing the impact of the interaction between fear and disease threat on the purchase intention of ultra-processed foods, thereby further confirming that the emotional state of high-pressure workers can influence their consumption intentions. The primary theoretical contribution of this study is to provide a systematic explanation of how the disease threat associated with high-pressure work, and its interaction with health perception and fear, influences the purchase intention of ultra-processed foods.

## 2 Theoretical derivation and literature review

### 2.1 Perceived threat theory

The Perceived Threat Theory is a theoretical framework that explains how individuals perceive, evaluate, and respond to health threats (39). This theory posits that an individual’s perception of a health threat influences their health-protective behaviors (40, 41). To strengthen the explanatory power of this framework, this study integrates it with two additional behavioral theories: the Health Belief Model (HBM) and the Theory of Planned Behavior (TPB). These theories provide complementary insights into how health threats translate into specific behavioral outcomes, such as the purchase intention of ultra-processed foods.

Perceived Threat Theory emphasizes that individuals’ behaviors are motivated by their perception of the severity of a health threat and their perceived susceptibility to it (40, 41). When individuals perceive a high level of threat, they are more likely to engage in protective behaviors. For instance, Bowling et al. (42) found that individuals with a high perceived threat of HIV infection were more likely to adopt safer sexual behaviors. Similarly, Beidler et al. (43) reported that women with a high perceived threat of breast cancer were more motivated to undergo regular mammograms. These findings align with the core idea of the Perceived Threat Theory, which emphasizes the role of threat perception in driving health-related decisions.

In the context of this study, perceived threat refers to individuals’ subjective assessment of the health risks associated with consuming ultra-processed foods. According to research, a higher perceived threat of diet-related diseases (e.g., obesity, diabetes) can motivate individuals to reduce their consumption of ultra-processed foods (44, 45).

The HBM provides a more nuanced understanding of how perceived threats influence health behaviors by introducing constructs such as perceived severity, susceptibility, benefits, and barriers (46). In the context of purchasing intentions for ultra-processed foods, the HBM helps explain how individuals weigh the perceived health risks (e.g., the threat of diet-related diseases) against the perceived benefits (e.g., taste, convenience) of consuming ultra-processed foods. For example, if an individual perceives the health threat of ultra-processed foods as severe but also perceives significant barriers to healthier alternatives (e.g., time, cost), they may still choose to purchase ultra-processed foods despite the perceived threat (47).

The TPB further strengthens the framework by emphasizing the role of attitudes, subjective norms, and perceived behavioral control in shaping intentions and behaviors (48). In this study, the TPB is used to explain how perceived threats influence the purchase intention of ultra-processed foods through these psychological pathways. Specifically, perceived threat may shape individuals’ attitudes toward ultra-processed foods, with higher perceived threats leading to more negative attitudes (28). Sociocultural influences, such as societal preferences for unhealthy foods or the stigma associated with healthy eating, may moderate the relationship between perceived threat and the intention to purchase ultra-processed foods (49). Individuals’ confidence in their ability to avoid ultra-processed foods (e.g., due to willpower or access to healthier alternatives) may also influence their purchase intentions (16).

Building on these integrated theories, this study proposes a precise mechanism linking perceived threat to the purchase intention of ultra-processed foods. Perceived Threat (from the Perceived Threat Theory) leads to heightened health awareness and perceived severity of diet-related diseases (50). These perceptions influence individuals’ attitudes and perceived behavioral control (from the TPB) (51), which in turn shape their purchase intentions for ultra-processed foods. The Health Belief Model moderates this relationship by considering individuals’ perceptions of the benefits and barriers to healthier choices.

The application of these theories to high-pressure work environments is particularly relevant, as stress and time constraints may exacerbate the demand for convenient food options, such as ultra-processed foods. Wang et al. (52) found that high-pressure workers with a high perceived threat of occupational diseases were more likely to adopt healthier dietary habits; however, the mechanisms underlying this behavior were not fully explored. This study aims to fill this gap by investigating how perceived threat, in combination with attitudes and perceived behavioral control, influences the purchase intention of ultra-processed foods among high-pressure workers.

### 2.2 Disease threat and purchase intention of ultra-processed foods

Disease, as a health threat, often leads consumers to take a series of coping measures closely related to consumer behavior (53, 54). In previous studies, researchers have found that disease threats increase consumers’ health awareness, making them more inclined to choose food options perceived as healthy and organic (55, 56). Although the purchasing behavior of ultra-processed foods differs significantly from that of wholesome organic foods, the decision-making process for both is similarly influenced by disease threats. In high-pressure work environments, threats to the health of high-pressure workers may stem from health risks in the work environment, concerns about personal health status, and reports of public health crises (57, 58). This disease threat prompts workers to seek behaviors that reduce health risks, such as decreasing the purchase of ultra-processed foods.

The reduced demand for ultra-processed foods among high-pressure workers may be due to disease threats triggering a series of psychological reactions, such as anxiety, worry, and fear, which increase the psychological burden and cognitive depletion of high-pressure workers (58, 59). For example, workers may reduce their purchase of ultra-processed foods due to concerns about the health risks associated with these products, thereby lowering their trust, satisfaction, and loyalty toward such foods (59, 60). In the long term, this disease threat may lead to workers’ sustained avoidance of ultra-processed foods, resulting in a lower purchase intention.

Based on the above analysis, this study proposes the following hypothesis:

*H1:* High-pressure workers with disease threat (High vs. Low) have a lower purchase intention of ultra-processed foods.

### 2.3 The mediating role of health perception

Health perception refers to an individual’s cognition and evaluation of their own health status, as well as the health attributes of products (61, 62). Among these, product health perception encompasses how consumers evaluate product ingredients, nutritional value, and safety, which significantly influences their purchasing decisions, loyalty, and judgments regarding product health factors (63, 64). Therefore, numerous researchers have conducted studies on how to enhance consumers’ health perception across various fields (65, 66). For instance, it has been found that health education can significantly improve patients’ health perception awareness (67, 68). However, this study did not focus on the health perception of high-pressure workers and their purchase intention of ultra-processed foods.

High-pressure workers often have a higher level of health perception (69). In previous studies, researchers have provided evidence on aspects such as work stress and physiological responses, immune issues, sleep problems, self-awareness and self-management, and work-life balance (70). Yet, the disease threats faced by high-pressure workers have rarely been explored in research. The work pressure and intense work environment faced by high-pressure workers increase their perception of disease threats, including the susceptibility and severity of diseases (71, 72). This enhances the behavioral motivation of high-pressure workers. A recent study found that consumers with a higher level of health perception are more inclined to purchase organic and green foods (73). However, the relationship between consumer health perception and the purchase intention of ultra-processed foods has not been thoroughly discussed. Furthermore, we need to explore how health perception mediates the relationship between the disease threat posed by high-pressure work and the purchase intention of ultra-processed foods. Specifically, the disease threat faced by high-pressure workers increases their perception of health, and this heightened health awareness prompts them to re-evaluate the risks associated with ultra-processed foods, leading to a reduced intention to purchase such products.

Based on the above analysis, this study proposes the following hypothesis:

*H2:* Health perception has a mediating effect on the relationship between the disease threat of high-pressure workers and their purchase intention of ultra-processed foods.

### 2.4 The moderating role of fear

Fear is a basic human emotion (74). In the field of psychology, the experience of fear encompasses multiple aspects, including neural mechanisms, emotional processing, and emotional regulation (75, 76). For instance, research in the realm of neural mechanisms indicates that fear processing involves several brain regions, which play crucial roles in the formation, storage, and retrieval of fear memories (77, 78). Moreover, the regulation of fear is also a topic of significant research interest (79, 80). For example, stimulation of the prefrontal cortex can enhance fear extinction memory, while vagus nerve stimulation has shown a nonlinear impact on fear extinction (81, 82). Fear not only affects an individual’s mental health but is also associated with various psychological disorders, such as post-traumatic stress disorder (83, 84). In particular, in high-pressure work environments, fear may be related to the perception of disease threat, thereby influencing an individual’s health perception and behavioral choices.

To provide a stronger theoretical basis for understanding the role of fear in consumer behavior, this study draws on the Terror Management Theory (TMT). TMT posits that the fear of existential threats, such as death, motivates individuals to seek meaning and security through cultural and self-esteem-related endeavors (85). According to TMT, individuals confronted with reminders of mortality (e.g., disease threats) exhibit heightened needs for self-protection and may engage in behaviors that reinforce their sense of meaning and control (86). This theory offers a robust framework for understanding how fear, particularly the fear of disease, influences consumer choices.

In previous studies, researchers have emphasized that an individual’s perception of disease can alter their psychological state, which in turn affects their behavior (87, 88). High-pressure workers, due to the significant stress of their jobs, may have a higher perception of disease threats. This perception, by triggering fear, further impacts their health behaviors (89). Specifically, fear may enhance high-pressure workers’ cognition of disease susceptibility and severity, thereby increasing their demand for and preference for health products (90). In the field of consumer behavior, the Theory of Planned Behavior posits that behavioral attitude, subjective norm, and perceived behavioral control collectively determine the intention to behave (91). We can speculate that fear, by enhancing the perception of disease threat, may influence the risk assessment of high-pressure workers regarding ultra-processed foods and their intention to purchase such products. Therefore, the combined effect of fear and disease threat on high-pressure workers reduces their intention to purchase ultra-processed foods.

Building on TMT, this study proposes that fear, particularly the fear of disease, drives individuals to seek meaning and reassurance through their consumption choices (36). In the context of ultra-processed foods, fear may lead to two possible outcomes. Individuals may choose to reduce their consumption of ultra-processed foods to mitigate the perceived health risks, thereby enhancing their sense of control and self-protection. Conversely, individuals may seek comfort or distraction from fear through impulsive or habitual consumption of ultra-processed foods (92), particularly if they perceive ultra-processed foods as convenient or pleasurable.

High-pressure work environments present a unique context in which fear and disease threat perception may interact to influence the purchase intention of ultra-processed foods. In such settings, stress and time constraints often exacerbate the demand for convenient food options, such as ultra-processed foods. However, when fear of disease-related risks is triggered, high-pressure workers may exhibit a mismatch between their intentions and actual behaviors. For instance, while fear may motivate individuals to prioritize healthier dietary choices, the pressures of their work environment may lead to impulsive or comfort-driven consumption of ultra-processed foods.

Based on the above analysis, this study proposes the following hypothesis:

*H3:* Fear has a moderating effect on the relationship between the disease threat perceived by high-pressure workers and their purchase intention of ultra-processed foods.

The theory, variables, and their models used in this study are presented in Table 1.

**Table 1:** Theory, variables and their models.

Theory	Variable	Variable definition	Theory definition	Relationship of variables	Theoretical framework
The Perceived Threat Theory	Disease threat	Disease threat refers to the serious adverse effects of diseases on human life and health.	Perceived Threat Theory highlights that individuals’ behaviors are motivated by their perceived severity of a health threat and their perceived susceptibility to it	Disease threat - Purchase intention	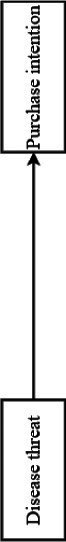
Health Belief Model	Health perception	Health perception refers to an individual’s subjective understanding and evaluation of their own health condition	The HBM provides a more nuanced understanding of how perceived threats influence health behaviors by introducing constructs such as perceived severity, susceptibility, benefits, and barriers	Disease threat - health perception - Purchase intention	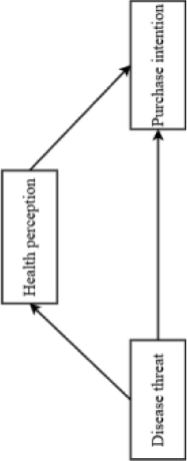
Terror Management Theory	Fear	Fear is a kind of strong repressed emotional experience of fear when people are facing some dangerous situation and trying to get rid of it but they can’t do anything about it	TMT posits that the fear of existential threats, such as death, motivates individuals to seek meaning and security through cultural and self-esteem-related endeavors (85)	Fear *disease threat - Purchase intention	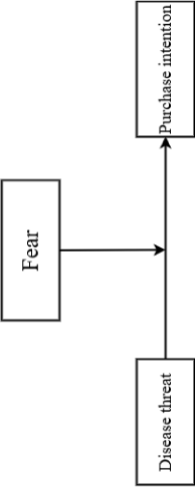
Theory of Planned Behavior	Purchase intention	Purchase intention refers to the probability that consumers are willing to take a specific purchase behavior. It is the subjective tendency formed by consumers’ attitude toward a product or brand and the effect of external factors.	The TPB further strengthens the framework by emphasizing the role of attitudes, subjective norms, and perceived behavioral control in shaping intentions and behaviors (48)		

**Figure 1:**
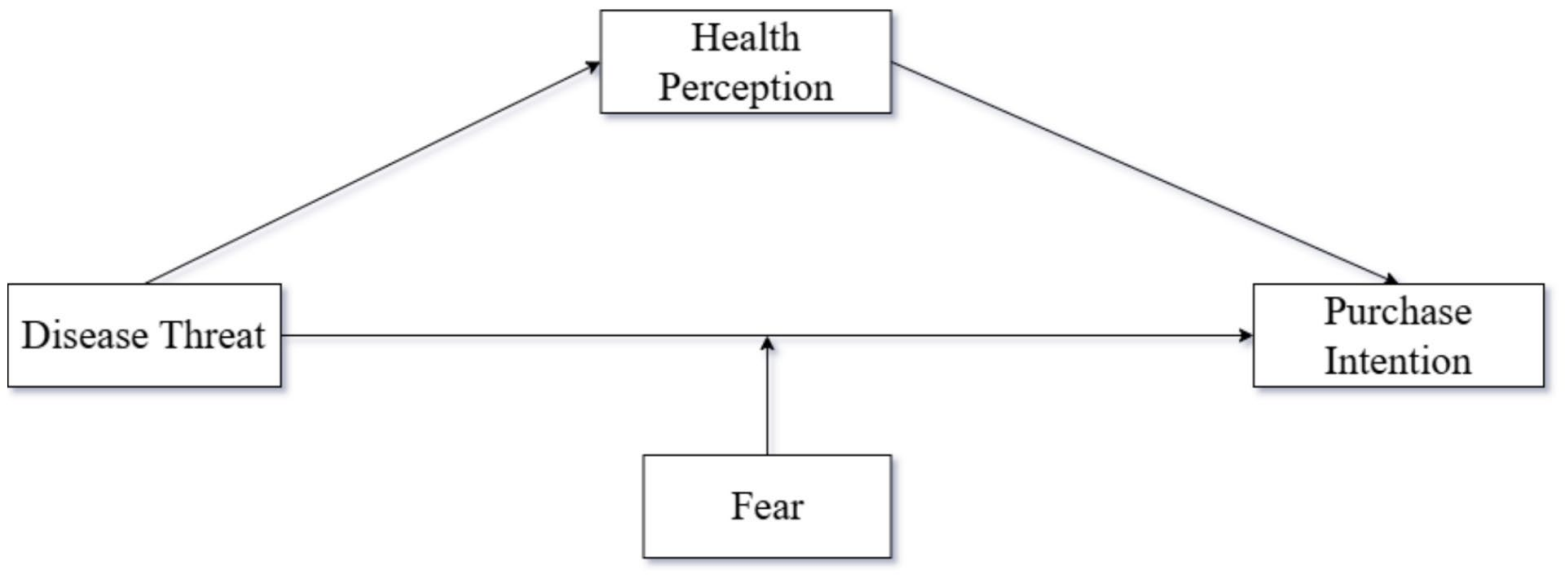
Experimental model diagram.

The conceptual model is shown in Figure 1.

## 3 Overview of the studies

In this study, the scenario experiment method was used to verify the above three hypotheses. It is a research method in psychology or behavioral science that combines virtual situation simulation with experimental control (92, 93). By constructing a realistic representative simulation scene, manipulating specific variables, and observing the behavior, decision or psychological reaction of participants in the situation, the causal relationship or theoretical hypothesis can be explored. To further test the above three hypotheses, we conducted three related studies. In Study 1, we examined the impact of disease threat (high vs. low) on the purchase intention of ultra-processed foods among high-pressure workers, testing Hypothesis H1. In Study 2, we analyzed the mediating role of health perception in the relationship between disease threat (high vs. low) and the purchase intention of ultra-processed foods among high-pressure workers, testing Hypothesis H2. In Study 3, we explored the moderating effect of fear (high vs. low) on the relationship between disease threat (high vs. low) and the purchase intention of ultra-processed foods among high-pressure workers, testing Hypothesis H3. To better manipulate the disease threat perceived by high-pressure workers, we used different textual stimuli in each study. For example, in Study 1, we used the monkeypox epidemic as the stimulus material. The demographic information for each study is presented in Table 2. The measurement topic of demographic information is referred to as the study of Liang et al. (94). The research framework for each study is shown in Table 3. The measurement items used in each study are provided in Table 4.

**Table 2:** Demographic information of the three experiments.

Variable	Items	Experiment 1 (*N* = 278)		Experiment 2 (*N* = 280)		Experiment 3 (*N* = 280)	
		Frequency	Proportion	Frequency	Proportion	Frequency	Proportion
Gender	Male	137	49.3%	141	50.4%	156	55.7%
	Female	141	50.7%	139	49.6%	124	44.3%
Age	18–25 years old	80	28.8%	73	26.1%	37	13.2%
	26–40 years old	73	26.3%	69	24.6%	110	39.3%
	41–60 years old	52	18.7%	72	25.7%	72	25.8%
	Over 61 years old	73	26.3%	66	23.6%	61	21.7%
Education background	Primary school	17	6.1%	14	5%	23	8.2%
	Junior high school	30	10.8%	33	11.8%	27	9.6%
	Technical secondary school, High school	43	15.5%	44	15.7%	45	16.1%
	Junior college	45	16.2%	42	15%	47	16.8%
	Undergraduate college	65	23.4%	73	26.1%	51	18.2%
	Postgraduate	60	21.6%	57	20.4%	70	25%
	Doctor-postgraduate	18	6.5%	17	6.1%	17	6.1%

**Table 3:** Experimental frameworks associated with the three studies.

Experiment	Experiment 1	Experiment 2	Experiment 3
Purpose	To test for main effect (H 1)	To test the mediation effect of health perception (H2)	To test the moderating effect of fear (H3)
Independent variable	Disease threat	Disease threat	Disease threat
Dependent variable	Purchase intention	Purchase intention	Purchase intention
Mediators	–	Health perception	–
Moderator	–	–	Fear
Methods	ANOVA	ANOVA	ANOVA
		PROCESS 4	PROCESS 1
Experimental group Stimulation material	Urgent Notice: Monkeypox outbreak once again constitutes a public health emergency of international concern. This is the second time monkeypox has posed a global health threat since the World Health Organization declared the monkeypox outbreak a PHEIC on July 23, 2022.	Urgent Notice: Recently, cases of dengue fever have been reported in the city, and the epidemic prevention and control situation is severe. The primary symptoms of the disease are the sudden onset of high fever, headache, generalized muscle and joint pain, and rash. In severe cases, there may be dengue hemorrhagic fever, or even shock or death.	Urgent notice: According to the World Health Organization, the current bird flu epidemic has appeared sporadic cases in many countries and regions, and there is a risk of further spread. Birds mainly transmit the virus, and humans are at risk from contact with infected birds or their secretions or feces.
Control group stimulation material	Urgent Notice: Due to the increasingly hot weather, water consumption among residents is increasing daily. The city will be without water from 9 AM to 12 PM tomorrow. Please prepare in time.	Urgent Notice: Due to the increasingly hot weather, the use of air conditioning is becoming more frequent, and the power supply is being gradually strained. The city will experience a power outage from 7:00 AM to 8:00 AM tomorrow to ensure the electricity needed for daily life is available.	Urgent Notice: Due to the closure of large pork farms in the city, pork supply is tight. We suggest that consumers rationally consume pork and increase their consumption of other meats.
Results	Supported H1	Supported H2	Supported H3

**Table 4:** Scales used in the three experiments.

Variable	Measurement items	Scale source
Demographic information	What is your gender?	
	What’s your age?	
	What is your educational background?	
Perceived life threat	After you read the above scenario content, do you agree that you feel your life is threatened?	Heir et al. (95)
Health perception	Do you agree that you are very concerned about your health?	Ware (97)
	Do you agree that you worry more about your health than others?	
	Do you agree that your health is the most essential thing in your life?	
Fear	Do you agree that the production techniques of super-processed foods may have a long-term negative impact on the environment?	Cox and Evans (99)
	Do you agree that it is risky to specialize in new food technologies too quickly?	
	Do you agree that the techniques used to produce super-processed foods may have negative long-term health effects?	
Consumption preference	Do you choose to buy regular ultra-processed foods out of habit?	Saba and Di Natale (98)
Purchase intention	When you understand the above disease threats, do you agree to be willing to buy ultra-processed food?	Rausch and Kopplin (96)

## 4 Experimental design

### 4.1 Experiment 1: the impact of disease threat on high-pressure workers and their purchase intention of ultra-processed foods

In Experiment 1, we designed a single-factor between-subjects experiment (disease threat: high vs. low) to explore the impact of disease threat (high vs. low) on the purchase intention of ultra-processed foods among high-pressure workers. We recruited 280 participants through the professional survey platform Credamo.^2^ Due to two participants not completing the questionnaire, we excluded the invalid questionnaires, resulting in a total of 278 valid questionnaires. We randomly assigned all participants to either the high disease threat group (*n* = 139) or the low disease threat group (*n* = 139).

Experimental Procedure. First, we asked all participants to imagine that they were shopping in a large shopping mall and introduced the meaning and scope of ultra-processed foods. Subsequently, we sent the latest notice about the monkeypox epidemic to the high-disease-threat group and the latest notice about the city-wide water outage to the low-disease-threat group. The content of the two notices is shown in Table 2. Immediately afterward, we measured the subject’s responses to questions regarding perceived life threat (95). Then, we asked participants about their purchase intention of ultra-processed foods (96). Finally, we collected demographic information related to the participants.

### 4.2 Experiment 2: the mediating role of health perception

Experiment 2 aimed to verify the mediating role of health perception in the relationship between disease threat (high vs. low) and the purchase intention of ultra-processed foods among high-pressure workers. We designed a single-factor between-subjects experiment and recruited 280 participants through the professional survey platform Credamo (see text footnote 2, respectively). We randomly assigned all participants to either the high-disease-threat group (*n* = 140) or the low-disease-threat group (*n* = 140).

Experimental Procedure. First, we asked all participants to imagine that they were shopping in a large shopping mall and introduced the meaning and scope of ultra-processed foods. Subsequently, we sent the latest notice about the dengue fever epidemic to the high-disease-threat group and the latest notice about the city-wide power outage to the low-disease-threat group. The content of the two notices is shown in Table 2. Immediately afterward, we measured the subject’s responses to questions regarding perceived life threat (95). Then, participants were required to answer questions measuring health perception, such as “Do you agree that you are very worried about your health status?” (1 = Strongly Disagree, 7 = Strongly Agree) (97). Immediately afterward, we asked participants about their purchase intentions for ultra-processed foods (96).

Given that the study by Saba and Di Natale (98) found consumption preference to be an important factor in enhancing purchase intention, we included purchase preference as a control variable. We asked participants questions measuring their purchase preference, such as “Do you choose to buy regular ultra-processed foods out of habit?” (1 = Strongly Disagree, 7 = Strongly Agree) (98). Finally, we collected demographic information related to the participants.

### 4.3 Experiment 3: the moderating role of fear

In Experiment 3, we designed a 2 (disease threat: high vs. low) × 2 (fear: high vs. low) factorial experiment. Experiment 3 aimed to verify the moderating effect of fear on the relationship between disease threat and the purchase intention of ultra-processed foods among high-pressure workers. We recruited 280 participants through the professional survey platform Credamo (see text footnote 2, respectively). We randomly assigned all participants to either the high-disease-threat group (*n* = 140) or the low-disease-threat group (*n* = 140).

Experimental Procedure. First, we asked all participants to imagine that they were shopping in a large shopping mall and introduced the meaning and scope of ultra-processed foods. Subsequently, we sent the latest notice about the avian influenza epidemic to the high-disease-threat group and the latest notice about the shortage of meat product supply to the low-disease-threat group. The content of the two notices is shown in Table 2. Immediately afterward, we measured the subject’s responses to questions regarding perceived life threat (95). Then, participants were required to answer questions measuring fear, such as “Do you agree that the production technology of ultra-processed foods may have long-term negative impacts on health?” (1 = Strongly Disagree, 7 = Strongly Agree) (99); immediately after, we asked participants about their purchase intention of ultra-processed foods (96).

## 5 Result

### 5.1 Experiment 1 result and discussion

#### 5.1.1 Manipulation inspection

We performed an independent sample t-test with disease threat as the grouping variable and perceived life threat as the test variable. The results showed that the higher the disease threat, the higher the perceived life threat (M _high disease threat_ = 4.57, SD _high disease threat_ = 1.663; M _low disease threat_ = 3.83, SD _low disease threat_ = 1.88, t = 3.514, *p* < 0.001). This indicates that the manipulation in this study was effective.

#### 5.1.2 Main effect test

We took disease threat as the independent variable and purchase intention as the dependent variable, and conducted a one-way ANOVA. The results showed that high-pressure workers with a low disease threat (*M* = 5.5, SD = 1.51) had a significantly higher purchase intention of ultra-processed foods than those with a high disease threat (*M* = 4.23, SD = 1.431), *F* (1, 276) = 52.067, *p* < 0.001, *η*^2^ = 0.159. Therefore, Hypothesis H1 was supported.

Relationship between age, education, and purchase intention. We conducted a one-way ANOVA with educational background and age as independent variables and purchase intention as the dependent variable. The results showed that age had no significant effect on the purchase intention of high-pressure workers [*F* (3, 274) = 1.103, *p* = 0.348]. The educational background had no significant effect on the purchase intention of high-pressure workers [*F* (5, 272) = 1.896, *p* = 0.095]. Therefore, the academic background and age of the high-pressure work group had no significant effect on purchase intention.

#### 5.1.3 Control variable analysis

Given that Kuang et al. (73) found gender to be an essential factor affecting purchase intention among high-pressure workers, we included gender as a control variable. We took gender as the independent variable and conducted a one-way ANOVA. The results showed that gender had no significant impact on the experimental results [*F* (1, 276) = 0.293, *p* = 0.589]. Therefore, gender had no alternative explanatory effect, further supporting Hypothesis H1.

In Experiment 1, we confirmed that the disease threat perceived by high-pressure workers significantly affects their purchase intention of ultra-processed foods. Specifically, compared with low disease threat, high-pressure workers with high disease threat had a significantly lower intention to purchase ultra-processed foods. Meanwhile, we verified that gender had no significant impact on the experimental results, enhancing the accuracy of the study. Although Experiment 1 had the above research findings, it did not analyze the internal mechanisms and boundary conditions between disease threat and the purchase intention of ultra-processed foods. Therefore, in Experiment 2, we introduced health perception as a mediating variable to analyze its mediating effect.

### 5.2 Experiment 2 result and discussion

Manipulation inspection. We performed an independent sample t-test with disease threat as the grouping variable and perceived life threat as the test variable. The results showed that high-pressure workers with a higher disease threat (*M* = 4.34, SD = 1.761) perceived a higher life threat than low-pressure workers with a lower disease threat (*M* = 3.69, SD = 1.85), *t* = 3.01, *p* = 0.003. Therefore, the manipulation was effective in this study.

#### 5.2.1 Main effect test

We took disease threat as the independent variable and purchase intention as the dependent variable, and conducted a one-way ANOVA. The experimental results showed that the purchase intention of ultra-processed foods (M _high-disease threat_ = 4.19, SD _high-disease threat_ = 1.227; M _low-disease threat_ = 5.56, SD _low-disease threat_ = 1.528), *F* (1, 278) = 69.309, *p* < 0.001, *η*^2^ = 0.2. This indicates that in high-pressure work environments, high-pressure workers with high disease threat have a lower purchase intention of ultra-processed foods. This result supports Hypothesis H1.

#### 5.2.2 Mediation effect test

**Figure 2:**
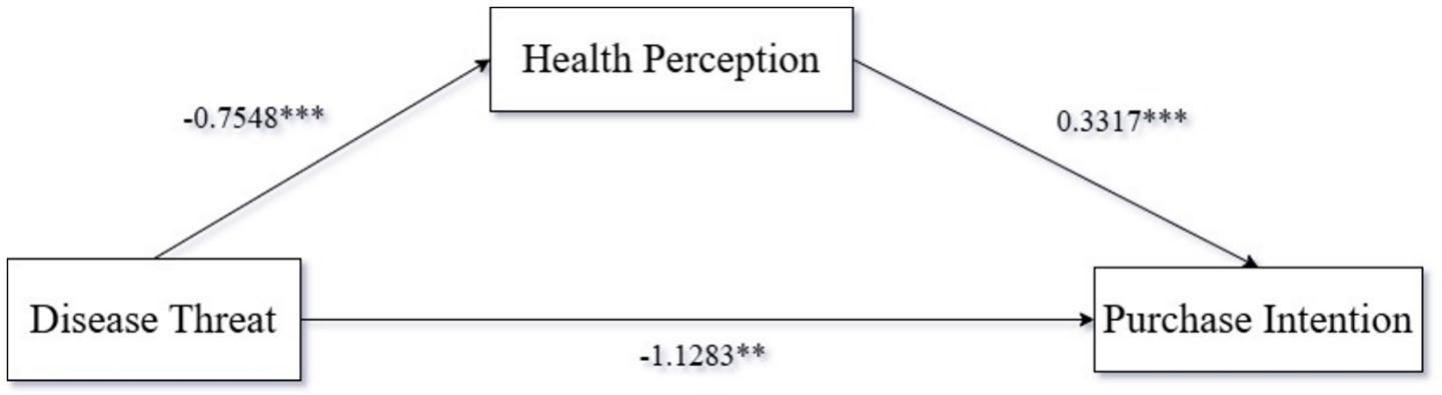
Mediating role of health perception.

We took disease threat as the independent variable, purchase intention as the dependent variable, and health perception as the mediating variable, and used Process Model 4 to analyze the mediating role of health perception [Bootstrap sample: 5000; (100)]. The experimental results showed that disease threat had a significant impact on purchase intention [*β* = −1.1283, *p* < 0.001, 95% CI = (−1.4589, −0.7976)]; disease threat had a significant impact on health perception [*β* = −0.7548, *p* < 0.001, 95% CI = (−1.024, −0.4848)]; health perception had a significant effect on purchase intention [*β* = 0.3317, *p* < 0.001, 95% CI = (0.1943, 0.469)]. Overall, the mediation effect of disease threat—health perception—purchase intention was significant [*β* = −0.2503, SE = 0.0711, 95% CI = (−0.4012, −0.1265)], as shown in Figure 2. Therefore, health perception has a significant mediating effect on the relationship between disease threat and purchase intention of ultra-processed foods among high-pressure workers, supporting Hypothesis H2.

#### 5.2.3 Control variable analysis

We included consumption preference as a covariate and conducted an ANCOVA (98). The results showed that consumption preference had no significant impact on the purchase intention of ultra-processed foods between the two disease threat groups [*F* (1, 278) = 7.161, *p* < 0.01]. Therefore, consumption preference had no significant effect on the experimental results, further supporting Hypothesis H1.

The relationship between purchase intention and gender, age, and education. We conducted a one-way ANOVA with gender, age, and educational background of the high-pressure work group as independent variables and purchase intention as the dependent variable. The results showed that gender had no significant effect on purchase intention [*F* (1, 278) = 0.674, *p* = 0.412]. Age had no significant effect on purchase intention [*F* (3, 276) = 0.157, *p* = 0.925]. Educational background had no significant effect on purchase intention [*F* (1, 278) = 0.674, *p* = 0.412]. Educational background had a significant impact on purchase intention [*F* (6, 273) = 6.416, *p* < 0.001]. It can be concluded that neither gender nor age has a significant effect on high-pressure work, but education does have a significant effect on high-pressure workers. To further enhance the robustness of the experiment, referring to the method of Tan et al. (101), we analyzed the effect of health perception on disease threat and purchase intention using educational background as a control variable. The results showed that educational background had no significant effect on the mediating effect of health perception [*β* = −0.2512, SE = 0.696, 95% CI = (−0.4021, −0.1287)].

Our findings revealed that health perception plays a significant mediating role in the relationship between disease threat and the purchase intention of ultra-processed foods among high-pressure workers. Specifically, the results showed that when high-pressure workers perceived a higher level of disease threat, their health perception was significantly enhanced. This heightened health perception, in turn, led to a reduction in their purchase intention of ultra-processed foods. The exclusion of consumption preference as a confounding variable in the experimental design allowed us to isolate and emphasize the specific influence of health perception in this context.

To further interpret the mediating effect of health perception, we consider its psychological and practical implications. High-pressure workers, often operating in stress-intensive environments, may perceive a heightened vulnerability to disease due to their demanding work conditions (102). This perceived vulnerability can trigger a heightened state of health awareness, as individuals become more attuned to potential health risks. The association between disease threat and health perception is deeply rooted in survival instincts, where individuals are motivated to avoid behaviors that may exacerbate health risks. In this case, ultra-processed foods, often linked to adverse health outcomes such as decreased immunity and increased risk of chronic diseases, become a focal point of avoidance behaviors.

The primacy of health perception over other factors, such as nutritional knowledge, can be attributed to its immediate and personal relevance. While nutritional knowledge provides a cognitive understanding of food choices (103), health perception taps into an individual’s emotional and psychological state, particularly in the context of disease threats. For example, high-pressure workers may rationally understand the nutritional benefits of healthier alternatives, but the fear of disease-related risks associated with ultra-processed foods consumption creates an emotional imperative to avoid such products. This aligns with the Terror Management Theory, which suggests that fear of existential threats (e.g., disease) drives individuals to seek coping mechanisms, such as healthier behaviors, to restore a sense of control and security.

Moreover, the interplay between disease threat and health perception can be intensified in high-pressure work environments. These environments often impose time constraints and stressors that narrow individuals’ cognitive bandwidth, making them more susceptible to fear-driven decision-making. When faced with the threat of disease, high-pressure workers may prioritize health-protective behaviors as a means of self-preservation, leading to a reduction in ultra-processed foods purchase intention. This behavior is further reinforced by the perceived consequences of ultra-processed foods consumption, such as immune system weakening, which aligns with the fear of disease exacerbation.

While this study did not explore moderating variables between disease threat and ultra-processed foods purchase intention, Experiment 3 introduced fear as a moderating variable to examine its interactive effect with disease threat. The findings suggest that fear amplifies the relationship between disease threat and health perception, further reducing ultra-processed foods purchase intention. This highlights the importance of considering psychological factors, such as fear, in understanding consumer behavior, particularly in high-pressure contexts.

### 5.3 Experiment 2 result and discussion

#### 5.3.1 Manipulation inspection

We performed an independent sample t-test with disease threat as the grouping variable and perceived life threat as the test variable. The results showed that the higher the disease threat, the higher the perceived life threat (M _high disease threat_ = 6.307, SD _high disease threat_ = 0.904; M _low disease threat_ = 5.692, SD _low disease threat_ = 1.77, *t* = 3.655, *p* < 0.001). This indicates that the manipulation in this study was effective.

#### 5.3.2 Main effect test

We took disease threat as the independent variable and purchase intention as the dependent variable, and conducted a one-way ANOVA. The results showed that high-pressure workers with low disease threat (*M* = 5.17, SD = 1.734) had a significantly higher purchase intention of ultra-processed foods than those with high disease threat (*M* = 4.4, SD = 1.413), *F* (1, 278) = 16.651, *p* < 0.001, *η*^2^ = 0.057. Therefore, Hypothesis H1 was supported.

Relationship between gender, age, education, and purchase intention. We conducted a one-way ANOVA with gender, education, and age as independent variables and purchase intention as the dependent variable. The results showed that gender had no significant effect on purchase intention [*F* (1, 278) = 0.114, *p* = 0.736]. The educational background had no significant effect on purchasing intention [*F* (6, 273) = 1.478, *p* = 0.186]. Age had no significant effect on purchase intention [*F* (3, 276) = 0.889, *p* = 0.447]. Therefore, gender, education, and age had no significant effect on purchase intention.

#### 5.3.3 Moderation effect test

**Figure 3:**
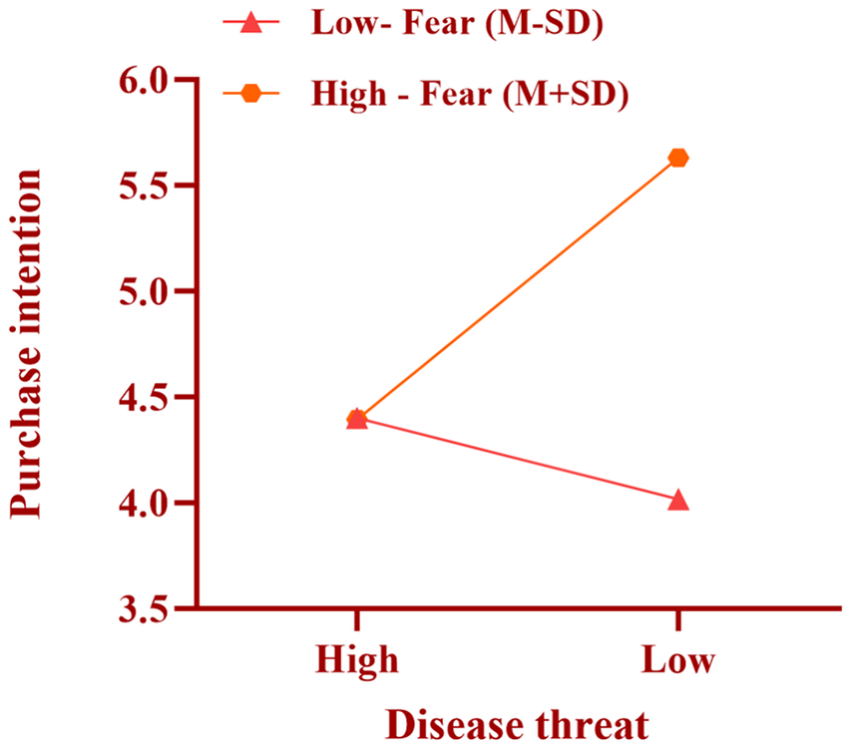
The interaction between fear and the threat of disease.

We took disease threat as the independent variable, purchase intention as the dependent variable, and fear as the moderating variable, and used Process Model 1 to analyze the moderating effect of fear [Bootstrap sample: 5000; (100)]. The experimental results showed that disease threat had a significant negative impact on purchase intention [*β* = −0.4259, *p* = 0.031, 95% CI = (−0.8141, −0.0377)]; fear had a significant negative impact on purchase intention [*β* = 0.3505, *p* < 0.001, 95% CI = (0.1704, 0.5306)]; the interaction between disease threat and fear had a significant negative impact on purchase intention [*β* = −0.7068, *p* < 0.001, 95% CI = (−1.0669, −0.3466)], as shown in Figure 3. Therefore, fear has a significant moderating effect on the relationship between disease threat and purchase intention of ultra-processed foods among high-pressure workers, supporting Hypothesis H3.

In Experiment 3, we investigated the significant mediating effect of fear on the relationship between the threat of disease and the willingness of hyperpressured workers to purchase ultra-processed food (H3). Specifically, the data showed that high-pressure workers with high fear levels were more likely to increase their consumption of ultra-processed foods under conditions of low disease threat. This suggests that the moderating effect of fear on consumption behavior is more significant when the disease threat is low. The effect of fear on consumption behavior was weaker in the high disease threat condition. This result suggests that the incremental effect of fear is relatively reduced when the disease threat is already high.

To gain insight into how fear mediates the relationship between disease threat and purchase intention, we further explored the psychological mechanisms underlying this relationship. According to the Health Belief Model and the Theory of Planned Behavior, fear influences an individual’s purchase behavior by enhancing their perception of health. Under low disease threat conditions, fear enhances individuals’ sensitivity to disease threats by increasing their perception of health risks, making high-pressure workers more inclined to avoid ultra-processed foods. In a high disease threat condition, where disease threat has already reached a high level, the moderating effect of fear is masked by health knowledge or environmental stress, so its effect is weakened.

## 6 Discussion

### 6.1 General conclusions

This study conducted three scenario experiments to verify the impact of disease threat on the purchase intention of high-pressure workers for ultra-processed foods and its internal mechanisms and boundary conditions. We found that in high-pressure work environments, workers with high disease threat tend to have a lower purchase intention of ultra-processed foods (H1). Health perception has a significant mediating effect on the relationship between disease threat and the purchase intention of ultra-processed foods (H2). Meanwhile, we found that fear has a significant moderating effect on the relationship between disease threat and the purchase intention of ultra-processed foods (H3). This not only addresses the shortcomings of previous research but also responds to the call to enhance the understanding of disease threats and consumer purchasing behavior in the context of the current epidemic (104, 105). In addition, this study focuses on the high-pressure working group, a consumer group that has increased sharply in recent years and often has high psychological pressure. Ultimately, this study further expands the application scope of the Perceived Threat Theory, providing valuable references for future research.

### 6.2 Theoretical significance and practical implications

This study demonstrates that heightened perceived disease threat among high-pressure workers significantly reduces their purchase intention for ultra-processed foods. This finding directly supports and extends the Perceived Threat Theory (106), which posits that cognitive appraisal of threats drives decision-making. While prior consumer psychology research focused on internally generated health concerns (107, 108), our work uniquely shifts the lens to the impact of external, objective environmental threats (e.g., pandemics, health crises) on the food choices of a vulnerable population. Crucially, we show that even when a strong intrinsic preference for ultra-processed foods exists (common under stress), the salience of external disease threats can effectively override this preference, suppressing ultra-processed foods purchase intentions. This significantly broadens the theoretical scope of Perceived Threat Theory by establishing a clear link between macro-environmental health risks and specific micro-level consumer food behaviors, filling a gap in understanding how contextual threats modulate established preferences.

The relationship between disease threat and reduced ultra-processed foods purchase intention is underpinned by the multidimensional nature of health perception. Our results indicate that increased disease threat elevates health consciousness (a key cognitive dimension of health perception), which subsequently drives the behavioral shift away from ultra-processed foods. This illuminates health perception’s critical mediating role in translating external threats into specific consumption avoidance behaviors, addressing a significant gap in research linking threat perception to concrete food purchasing actions. Furthermore, a pivotal theoretical contribution lies in identifying fear as a potent moderator, grounded in Terror Management Theory. High levels of fear, co-occurring with perceived threat, act as a powerful psychological driver, intensifying the avoidance of ultra-processed foods as a defensive mechanism to manage mortality anxiety. Conversely, low fear attenuates the threat’s impact. This represents a novel application of Terror Management Theory beyond conspicuous consumption, revealing how existential anxieties shape protective health behaviors like risk-averse food choices, and underscores the critical interplay between cognitive threat appraisal and intense affective responses in health decision-making.

The context of disease threat also appears to dynamically shape individuals’ knowledge structures regarding ultra-processed foods. Heightened threat and associated health concerns can trigger the formation of a more elaborate health knowledge structure. This enhanced cognitive framework enables finer discrimination of health risks associated with ultra-processed foods, facilitating more deliberate behavioral adjustments. This dynamic view of knowledge structure formation under threat provides a micro-foundation for understanding consumer information processing in risk-laden contexts. These interconnected findings hold substantial practical significance. For public health, they validate the efficacy of targeted risk communication leveraging environmental threat salience and calibrated fear appeals to promote healthier eating among high-pressure groups. Concurrently, they advocate for creating supportive environments.

For practice, these insights compel the food industry to proactively address shifting consumer risk perceptions through product reformulation (reducing health risks), transparent communication during crises, and targeted segmentation acknowledging heterogeneity in fear responses. Investments in employee wellness can also mitigate negative health associations. Ultimately, this study provides a robust theoretical framework explaining how environmental threats, filtered through cognitive and affective processes, shape UPF consumption among vulnerable populations, offering actionable guidance for health promotion and industry adaptation.

### 6.3 Policy implications

To address the rapid growth of high-pressure workers and the health challenges they face, the government should develop and implement targeted public health policies. First, governments can educate high-pressure workers about the health risks associated with ultra-processed food consumption through targeted public information campaigns, particularly in the context of disease threats and vulnerability to immunity. Such awareness campaigns can be combined with visual AIDS such as infographics and short videos to visually demonstrate the relationship between ultra-processed food consumption and reduced immunity. Secondly, the government should strengthen its supervision of food labeling and require clear health warnings on ultra-processed food products, particularly regarding immune function and the risk of chronic diseases. In addition, governments could provide tax incentives to encourage companies to produce and market healthier food options, such as ultra-processed foods with reduced sugar or increased dietary fiber, thereby reducing the price burden of healthy foods and making them more accessible to high-pressure workers.

Food companies should actively respond to policy guidance by developing healthier alternatives to ultra-processed foods and promoting their health benefits through science-based labels. For example, companies could market snack products that claim to “boost immunity” or develop “healthy convenience foods” with health claims on packaging, such as being “high in fiber” or “low in added sugar.” In addition, companies can use fear appeals for precision marketing to highlight the health risks associated with traditional ultra-processed foods while emphasizing the benefits of new health products. For example, the advertising slogan “Protect your immunity with healthy choices” can effectively guide consumers to choose healthier products. At the same time, companies can help employees reduce their reliance on ultra-processed foods by enhancing employee wellness programs, such as offering healthy food options in high-pressure work environments, providing nutrition education, and offering mental health support.

To achieve long-term impact, public-private partnerships that integrate the resources of governments, industry, and public health organizations are needed. For example, a collaborative or support program for “Healthy Eating Options” for high-stress workers could provide free or reduced-price healthy food options to employees in specific industries who are under high stress. Additionally, the development of consumer-friendly digital tools, such as mobile apps and websites, is crucial. These platforms can provide health risk assessments, immunity-boosting recipes or personalized dietary advice to help consumers make more informed food choices. Finally, future research and policy formulation should focus on the effectiveness evaluation of behavioral interventions and the adaptability of health strategies in different cultural and regional contexts to ensure the accurate implementation of policies and practices.

### 6.4 Research limitations

Despite the above research findings, this study still has several research shortcomings. Firstly, this study only explored the impact of the social epidemic environment on the purchase intention of ultra-processed foods, and whether the conclusions apply to the disease threat elements carried by ultra-processed foods themselves is worth further exploration. Secondly, the research subjects of this study only focused on the group of high-pressure workers, and the purchase intention of other consumer groups has not been further discussed. Finally, this study only focused on the subjective factors of consumers’ health perception and fear, and did not include objective factors for discussion. Future research can incorporate additional food elements and consumer environment factors, such as food nutrition, peer support, and other relevant variables.

More importantly, this study may have had a potential methodological bias, namely, social desirability bias. Specifically, participants may have responded to questions about their ultra-processed food consumption habits without honestly reporting their actual consumption behavior due to social norms or health awareness. This bias may have biased the results and reduced the credibility of the findings. Therefore, future research is recommended to employ more objective data sources, such as actual purchase records or consumer sales data, to validate the findings of this study. In addition, future research can further explore the impact of food nutrition, consumption environment (such as spousal support and workplace policies), and cultural differences on consumption behavior to enrich the theoretical framework and enhance the external validity of the research.

## Data Availability

The original contributions presented in the study are included in the article/supplementary material, further inquiries can be directed to the corresponding author/s.

## Footnotes

^1^ https://news.cctv.com/2024/11/23/ARTIJK2a7ojkS9LWWtFXs5DE241123.shtml

^2^ https://www.credamo.com/
